# SIUMB recommendations for focal pancreatic lesions

**DOI:** 10.1007/s40477-020-00522-2

**Published:** 2020-09-04

**Authors:** Mirko D’Onofrio, Ilario de Sio, Paoletta Mirk, Gianpaolo Vidili, Michele Bertolotto, Vito Cantisani, Cosima Schiavone, The SIUMB experts committee, Esterita Accogli, Fabia Attili, Raffaella Basilico, Michele Bertolotto, M. Gabriella Brizi, Elisabetta Buscarini, Corrado Caiazzo, Fabrizio Calliada, Vito Cantisani, Alessandro Carriero, Alder Casadei, Orlando Catalano, Gaspare D’Anneo, Marco De Prizio, Ilario de Sio, Giulio Di Candio, Mirko D’Onofrio, Ferdinando Draghi, Francesco Maria Drudi, Giovanna Ferraioli, Giampiero Francica, Antonio Granata, Giovanni Iannetti, Giovanni Maconi, Fabrizio Magnolfi, Paoletta Mirk, Fabio Piscaglia, Maurizio Pompili, Gian Ludovico Rapaccini, Cosima Schiavone, Luca Maria Sconfienza, Carla Serra, Maurizio Soresi, Stefania Speca, Roberto Stramare, Luciano Tarantino, Massimo Valentino, Gianfranco Vallone, Gianpaolo Vidili

**Affiliations:** 1 Department of Radiology, G.B. Rossi Hospital, University of Verona, Verona, Italy; 2 Department of Hepatogastroenterology, Università degli Studi della Campania Luigi Vanvitelli, Naples, Italy; 3 Department of Radiology, Catholic University of the Sacred Heart-Fondazione Policlinico A. Gemelli, Rome, Italy; 4 Department of Medical, Surgical and Experimental Sciences, University of Sassari, Viale San Pietro 43b, 07100 Sassari, Italy; 5 Department of Radiology, University of Trieste, Ospedale di Cattinara, Trieste, Italy; 6 Department of Radiological Sciences, Policlinico Umberto I, University La Sapienza, Rome, Italy; 7 Unit of Ultrasound in Internal Medicine, Department of Medicine and Science of Aging, “G. d’Annunzio” University of Chieti, Chieti, Italy

**Keywords:** Pancreas, Ultrasound, Pancreatic cancer, Pancreatic cystic lesions, Contrast-enhanced ultrasound, CEUS, Pancreatic biopsy, Pancreatic cancer, Pancreatic adenocarcinoma

## Abstract

Contrast-enhanced ultrasonography (CEUS) is increasingly being performed in Italy and Europe, particularly in the field of hepato-gastroenterology. Initially, it was mainly carried out to characterize focal hepatic lesions, but, since then, numerous studies have demonstrated its efficacy in the differential diagnosis of focal pancreatic pathologies (D’Onofrio et al. in Expert Rev Med Devices 7(2):257–273, 2010; Vidili et al. in J Ultrasound 22(1):41–51, 2019). The purpose of this paper is to provide Italian Medical Doctors with recommendations and thereby practical guidelines on the management of these patients. The present paper reports the final conclusions reached by the SIUMB guideline commission. This paper addresses particularly percutaneous ultrasound (US) examination (transabdominal US) and is drawn up specifically for publication.

## Background

Contrast-enhanced ultrasonography (CEUS) is increasingly being performed in Italy and Europe, particularly in the field of hepato-gastroenterology. Initially, it was mainly carried out to characterize focal hepatic lesions, but, since then, numerous studies have demonstrated its efficacy in the differential diagnosis of focal pancreatic pathologies [1, 2]. The purpose of this paper is to provide Italian Medical Doctors with recommendations and thereby practical guidelines on the management of these patients. The present paper reports the final conclusions reached by the SIUMB guideline commission.

A bibliographic search for focal pancreatic lesions was carried out on PubMed using the following search terms: 2006–2016:

- "contrast enhanced ultrasound".
- "pancreatic cancers".
- "pancreatic adenocarcinoma"/"adenocarcinoma".
- "pancreatic neuroendocrine tumors"/"endocrine tumors".
- "pancreatic cystic lesions"/"cystic lesions".

The search led to the identification of 879 articles. Activating a filter for clinical trials, reviews, and meta-analyses, the number was reduced to 397 articles.

We proceeded with filtering these papers by including only:

- studies conducted on humans;
- studies in which the performance of CEUS in the characterization of pancreatic lesions was evaluated and sensitivity/specificity or positive/negative predictive values (PPV/NPV) were reported;
- studies in which Sonovue was used (data related to Sonazoid and Definity were excluded, as these contrast agents are not used in Italy);
- studies providing a qualitative evaluation of the contrast agent (we excluded studies providing a quantitative evaluation based on wash in/wash out times or image interpretation using software such as Photoshop etc.);
- studies of at least 30 patients (at least 10 benign pancreatic lesions and 10 malignant pancreatic lesions);
- studies published in English;
- studies in which histology, computed tomography (CT), and/or magnetic resonance imaging (MRI) and/or clinical examination (clinical–radiological follow-up) were considered as the gold standard.

This further skimming led to the evaluation of 1 clinical trial, 3 reviews, and 2 meta-analyses. The document produced on the basis of the selected studies was presented to a panel of experts gathered on 17 November 2019 to discuss and vote on each single point/recommendation.

This paper addresses particularly percutaneous ultrasound (US) examination (transabdominal US) and is drawn up specifically for publication.

## Pancreas

When a pancreatic lesion is detected, an immediate differential diagnosis is essential to proceed with appropriate management. Incidental detection of a pancreatic lesion occurs quite frequently in some countries (e.g., the United States).

Focal pancreatic lesions identified at the conventional US examination can be studied by CEUS to improve tissue characterization: solid pancreatic lesion vs cystic pancreatic lesion [1].

### Approval 84%

Although the role of elastosonography is not yet well defined, its use in pancreatic lesions to improve the management is reported in the literature [3].

After US detection of one or more focal cystic lesions of the pancreas, the patient should be submitted directly to MRI as a second-line examination to obtain further diagnostic information.

## US techniques

### Contrast-enhanced ultrasonography (CEUS)

Focal pancreatic lesions detected at conventional US examination can be further studied using CEUS for the following diagnostic purposes [4]:

1. Characterization of ductal adenocarcinoma (LoE 1b, GoR A).
2. Differential diagnosis between pseudocysts and cystic pancreatic cancer (LoE 1b, GoR A).
3. The ability to differentiate vascularized (solid) components from non-vascularized (liquid-necrotic) components within a lesion (LoE 1b, GoR A).
4. The best definition of the size and margins of the lesion, including its relationship with the adjacent vessels (LoE 2b, GoR B).
5. Management of the diagnostic work-up of the lesion aiming at the best possible distinction between solid and cystic lesions, thus obtaining information to guide the choice of the most appropriate subsequent diagnostic investigation (LoE 5, GoR C).
6. Diagnosis if CT outcome is uncertain or inconclusive (vascularization of solid pancreatic lesions; differential diagnosis between pseudocysts and pancreatic cystic neoplasms, particularly mucinous cystic neoplasms) (LoE 5, GoR C).

### Approval > 90%

#### Contrast-enhanced endoscopic US (CE-EUS)

CE-EUS can be recommended to [4]:

1. Improve characterization of pancreatic ductal adenocarcinoma on the basis of the hypovascular pattern mainly using CE-EUS and color Doppler (LoE 1b, GoR A).
2. Discriminate between chronic mass-forming pancreatitis and ductal adenocarcinoma in patients with chronic pancreatitis (LoE 1a, GoR A).
3. Improve discrimination between pancreatic cystic tumors and pancreatic pseudocysts (LoE 1b, GoR A).

### Approval > 90%

#### Elastosonography

1. Endoscopic US (EUS) elastography is useful as a complementary tool in the characterization of focal pancreatic lesions [3].
2. If there is a strong clinical suspicion of pancreatic cancer in the presence of inconclusive or negative biopsy outcome, detection of a "hard" focal lesion at elastography, and/or a suspicious mass indicating neoplasia at CE-EUS (hypovascular lesion), this should guide the clinical management suggesting a repeated EUS fine needle aspiration (FNA) or referring the patient directly to surgery [3, 5].
3. At the present time, EUS elastography cannot be recommended for differentiation between advanced chronic pancreatitis and pancreatic ductal adenocarcinoma as both diseases cause similar tissue "hardness" in an elevated number of cases [3].

### Approval 100%

#### Focal pancreatic lesions

##### Pancreatic adenocarcinoma

- CEUS can be a first-line diagnostic tool in the characterization of pancreatic ductal adenocarcinoma.
- In contrast-enhanced examinations, pancreatic adenocarcinoma shows a typical poor enhancement due to a strong intralesional desmoplastic reaction, which causes low mean vascular density and very poor perfusion.
- In patients with an easily explored pancreatic region, sensitivity of CEUS in the diagnosis of ductal pancreatic adenocarcinoma is adequate and does not differ statistically from that of contrast-enhanced CT. Comparison of diagnostic sensitivity of CEUS and contrast-enhanced CT reported in the literature shows that there is no statistically significant difference between the sensitivity of the two techniques. CEUS and contrast-enhanced CT provide the same possibility of diagnosing pancreatic adenocarcinoma. The combination of CEUS and contrast-enhanced CT can increase diagnostic performance and provide an early diagnosis of ductal adenocarcinoma.
- US/CEUS limitations are not linked to the size of the tumor, but to the technique and the patient’s condition including meteorism and constitutional factors.
- CEUS is a safe and feasible method providing an immediate and more accurate characterization of pancreatic lesions and possible liver metastases detection.
- In a meta-analysis, the overall sensitivity (0.89 [95% CI 0.85–0.92]), specificity (0.84 [95% CI 0.77–0.89]), and diagnostic odds ratio (DOR) (61.12 [95% CI 34.81–107.32]) highlighted the outstanding ability of CEUS in the characterization and distinction between adenocarcinoma and other pancreatic pathologies [6].
- All lesions appearing solid at conventional US and hypovascular at CEUS should be considered ductal adenocarcinomas until proven otherwise.

### Approval 100%

- The results reported in the literature confirm the reliability of US in case a small pancreatic nodule is detected and characterized by CEUS as adenocarcinoma, even if subsequent CT outcome is negative. The finding should be assessed using MRI or EUS. Thus, if a small pancreatic nodule is incidentally detected at US imaging with or without contrast enhancement, the patient should be referred directly to MRI to obtain a prompt diagnosis of ductal adenocarcinoma.
- CEUS is a sensitive method for detecting liver metastases and can be decisive in case of small liver lesions.

### Approval > 95%

According to the recent guidelines, CEUS may become the first-line diagnostic tool in the characterization of pancreatic ductal adenocarcinoma detected by means of ultrasound [4]. The main advantage of CEUS over other diagnostic methods is the possibility of obtaining a real time dynamic study of the pancreatic gland by using blood pool contrast agent [7]. The study procedure is focused on real-time evaluation of a pancreatic lesion after US contrast injection. In particular, an arterial phase can be observed, which has a duration of 10–30 s followed by a venous phase lasting from 30 to 120 s. In contrast-enhanced examinations, pancreatic adenocarcinoma shows a typical poor enhancement due to a strong intralesional desmoplastic reaction, which causes low mean vascular density and a very poor perfusion [8]. In a study, D'Onofrio et al. compared diagnostic sensitivity of US/CEUS and contrast-enhanced CT. They found that there is no statistically significant difference between the sensitivity of the two techniques (*p* = 0.678), i.e., CEUS and contrast-enhanced CT provide the same possibility of diagnosing pancreatic adenocarcinoma. However, some discordant points between the two methods emerged from this study: 9.77% of the total patient population, particularly patients with small pancreatic lesions (4 lesions out of 13 measured < 2 cm; 7 lesions out of 13 measured 2–3 cm), were diagnosed only at US/CEUS. These data are in agreement with the current scientific literature, which shows that 10% of pancreatic ductal adenocarcinomas are isodense compared to the surrounding gland, and moreover, that the prevalence of isodense tumors on contrast-enhanced CT is greater in lesions < 2 cm [9, 10]. In view of these conclusions, it is clear that the combination of US/CEUS and contrast-enhanced CT can increase diagnostic accuracy and provide an early diagnosis of ductal adenocarcinoma. On the other hand, 9 lesions were diagnosed only at contrast-enhanced CT: in 2 cases, US/CEUS did not identify focal lesions, and in 7 cases, US/CEUS showed different echoic features compared to the typical enhancement pattern, and CT showed 7 lesions > 3 cm. US/CEUS limitations are not linked to the size of the tumor, but to the technique and the patient’s condition including meteorism and constitutional causes. All this confirms that the combination of US/CEUS and contrast-enhanced CT contributes to more accurate diagnostic results. In conclusion, in patients with an easily explored pancreatic region, sensitivity of US/CEUS in the diagnosis of ductal pancreatic adenocarcinoma is adequate and does not differ statistically from that of contrast-enhanced CT. US/CEUS sensitivity seems to be higher in small- and medium-sized lesions, whereas contrast-enhanced CT sensitivity is higher in larger lesions. A combination of the two methods can thus provide a greater diagnostic accuracy in patients with pancreatic ductal adenocarcinoma [11].

CEUS has proved to be an accurate imaging method for evaluating the vascularity of pancreatic lesions and in the differentiation between solid and cystic lesions, thereby influencing the choice of subsequent diagnostic investigations in order to obtain a useful, immediate, and faster diagnosis [3, 11, 12]. In pancreatic ductal adenocarcinoma, a rapid diagnosis is very important. Although CEUS is relatively new in the evaluation of the pancreas, it is a safe and feasible technique which provides a better and immediate characterization of the lesion and permits staging of the neoplasm during US examination [13]. The primary objective of the meta-analysis carried out by D’Onofrio et al. was to evaluate the diagnostic ability of CEUS to identify and characterize typically hypovascularized pancreatic ductal adenocarcinomas. The merit of CEUS lies mainly in the ability to differentiate pancreatic adenocarcinoma from other masses of a different etiology but with a similar appearance on conventional US image, thanks to the dynamic evaluation allowing a direct observation of the post-contrast agent impregnation pattern, suggesting other possible diagnoses in case of isovascularized or hypervascularized lesions. In this meta-analysis, the overall sensitivity (0.89 [95% CI 0.85–0.92]), specificity (0.84 [95% CI 0.77–0.89]), and diagnostic odds ratio (DOR) (61.12 [95% CI 34.81–107.32]) support the ability of CEUS to characterize and distinguish between adenocarcinoma and other pancreatic pathologies, thus allowing a correct management of the pathology. CEUS should, therefore, be performed immediately after the conventional US if a pancreatic mass is detected. Another important step in the characterization of a pancreatic neoplastic mass is distinction between a solid lesion and a cystic lesion. The role of CEUS in determining the nature of a pancreatic lesion detected at conventional US is supported by the results of the meta-analysis showing sensitivity 0.95 (95% CI 0.93–0.96), specificity 0.72 (95% CI 0.58–0.83), and DOR 57.63 (95% CI 33.62–98.78). Particularly in the further investigation of pancreatic cystic lesions, CEUS improves the differential diagnosis between non-neoplastic cystic lesions (pseudocysts with a typically avascular content) and neoplastic lesions (such as mucinous cystadenoma) by accurately depicting the post-contrast agent impregnation of septa and nodules as well as the intralesional tissues in general. CEUS and CE-EUS can be used for a correct characterization of pancreatic lesions. However, in further investigation of incidentally detected pancreatic masses, CEUS improves the accuracy of conventional US providing a faster diagnosis and a better management of the disease. CEUS (transabdominal approach) and CE-EUS (endoscopic approach) should, therefore, be carried out for a more accurate evaluation of the pancreatic masses as this may benefit the clinical management of the patients [6].

In a study published by D’Onofrio et al., a comparison of CEUS and contrast-enhanced CT showed that the outcome was similar in the characterization of ductal adenocarcinoma. It should be pointed out that the study included only patients whose pancreatic gland was visible at US and in whom conventional US had already identified a solid lesion which required further characterization using CEUS. There was no statistically significant difference between CEUS and CT in the characterization of ductal adenocarcinoma. However, only CEUS did not yield false-negative results, whereas CT yielded false-negative results in the detection and characterization of the lesions, particularly smaller lesions < 2 cm [11]. In agreement with the scientific literature and our previous experience, detection of a small pancreatic adenocarcinoma is difficult at CT in the absence of mass effect. In conclusion, the results obtained in this study, which are in agreement with the data reported in the literature, support the credibility of US if a small pancreatic nodule is detected and characterized as adenocarcinoma at CEUS, also in the presence of a subsequent negative outcome of CT. In that case, the lesion requires further investigation using MRI or EUS. Therefore, if a small pancreatic nodule is incidentally detected at US examination, the patient should be referred directly to MRI to obtain the benefit of a fast diagnosis of pancreatic ductal adenocarcinoma.

In a large multicenter study of 1439 pancreatic lesions, D’Onofrio et al. correctly characterized solid pancreatic lesions reaching an accuracy of 91.7%. Particularly, pancreatic ductal adenocarcinoma was correctly characterized reaching an accuracy of 87.7% on the basis of hypovascular appearance at CEUS. This means that all solid lesions detected at conventional US and appearing hypovascular at CEUS should be considered ductal adenocarcinomas until proven otherwise. In other series of histologically proven ductal adenocarcinomas including more than 50 cases, ductal adenocarcinoma was reported to be hypovascular in 73–93% of cases [14–16]. CEUS outcome was also accurate in demonstrating hypervascularization of endocrine tumors.

In a multicenter study (PAMUS, see below), the accuracy of CEUS in characterizing neuroendocrine tumors was 90.5%. Also other interesting data are reported regarding the characterization of cystic lesions, as cystic tumors and pseudocysts were correctly diagnosed by CEUS reaching an accuracy of 97.1% and 99%, respectively. In this study, the highest level of accuracy of CEUS compared to conventional US was thus achieved in the characterization of pancreatic lesions, showing a statistically significant difference (*p* < 0.0001) in the relative receiver-operating characteristic (ROC) curves. In conclusion, CEUS can increase the accuracy of US in the study of incidentally detected pancreatic masses, thus leading to a faster diagnosis. CEUS can acquire also other roles that may become the objects of further investigation: first, it can contribute to a better management of lesions detected at the conventional US examination; second, it can become a problem-solving method [17].

US is the first-line imaging method used in patients with symptoms suggesting malignant pathology of the pancreas. However, US sensitivity is markedly reduced in very small tumors, and the use of US contrast agent does not improve the detection of pancreatic nodules depending on gland exploration and nodule size. On the other hand, the use of US contrast agent improves diagnostic power of the method by allowing a highly accurate characterization of ductal adenocarcinoma.[16]. Finally, in addition to determining local spread of the tumor using B-mode US and color-Doppler US evaluation, which should always be performed before CEUS, the method can detect liver metastases and be decisive in case of small liver lesions [18, 19].

#### Neuroendocrine tumors

- CEUS is significantly more accurate than the conventional US in the diagnosis of nonfunctional neuroendocrine tumors of the pancreas [20].
- CEUS is more sensitive than the conventional US in identifying liver metastases caused by neuroendocrine cancer.

### Approval 100%

US combined with CT and/or MR cholangiopancreatography (MRCP) is recommended. The decision to carry out CT or MRI depends on the preference, skill, and experience of the radiologist and the availability of equipment in the individual institutions. Somatostatin receptor scintigraphy has so far been indicated as the main single screening method in the assessment of extrahepatic localizations. However, positron emission tomography (PET)-CT with Ga^68^ and F^18^-DOPA seems to be an interesting method able to provide a better resolution and detect more lesions. Patients with small nonfunctional neuroendocrine tumors of the pancreas should undergo EUS, as EUS-guided FNA biopsy has yielded good results in confirming the diagnosis. CEUS seems to improve the characterization of liver metastases caused by neuroendocrine tumors, and CE-EUS could prove effective in characterizing pancreatic endocrine tumors [20].

CEUS is significantly more accurate than the traditional B-mode US in the diagnosis of nonfunctional neuroendocrine tumors of the pancreas, showing correlation between CEUS enhancement pattern and Ki67 index [20, 21]. In addition, CEUS is more sensitive than the traditional US in identifying liver metastases caused by neuroendocrine cancer; these lesions appear at CEUS as irregularly hypervascularized lesions [20, 22].

## Pancreatic cystic lesions

Italian guidelines on cystic tumors [23]

1. Conventional US of the pancreas cannot provide a definitive diagnosis of pancreatic cystic neoplasms (LoE 5, GoR D).
2. There are not enough detailed data in the literature regarding the use of CEUS in the differentiation between mucinous and non-mucinous pancreatic cystic lesions (LoE 5, GoR C).
3. MRI and contrast-enhanced CT are the first-line diagnostic techniques for differentiating between benign and malignant pancreatic cystic neoplasms. The performance of CEUS is similar to that of MRI and contrast-enhanced CT in pancreatic cystic lesions which are visible at US examination (LoE 1b, GoR).
4. There are no data in the literature supporting the role of percutaneous US-guided biopsy of pancreatic cystic neoplasms. FNA of these lesions should be carried out using the EUS approach (LoE 5, GoR D).
5. The role of each imaging method in the follow-up of asymptomatic patients with pancreatic cystic neoplasms depends on the size and number of lesions.
6. Small single cystic lesions (< 1 cm) visible on US image: US should be preferred until a change in size. If this occurs, CEUS or MRI should be performed to evaluate the presence of "suspicious" features. MRI with MRCP alternating with US should be used to evaluate the development of new cystic neoplasms of the pancreas. If MRI shows new cystic lesions, follow-up should be continued using MRI.
7. Small single cystic lesions (< 1 cm) that are not visible on US image: MR/MRCP (LoE 5, GoR D).
8. Large cystic lesions (≥ 1 cm) visible on US image: US should be preferred until a change in size. If this occurs, CEUS or MRI should be performed to evaluate the presence of "suspicious" features (dimensions, nodules, septa, contents, morphology). MRI with MRCP alternating with US is currently performed to evaluate the development of new cystic neoplasms.
9. Large cystic lesions (≥ 1 cm) not visible on US image: MRI with MRCP or contrast-enhanced CT. In patients requiring close follow-up (3 months), contrast-enhanced CT should be performed in elderly patients only in the absence of renal insufficiency and/or in patients with absolute contraindications to MRI (LoE 5, GoR D).
10. Multiple cysts: MRI with CPRM (LoE 5, GoR D).

### Approval 100%

- In the presence of pancreatic cystic lesions easily explorable by US, the results of CEUS and MRI reported in the literature in detecting intralesional nodules and septa are very similar.
- CEUS should be considered a complementary imaging method in the characterization of pancreatic cystic masses detected at abdominal conventional US, and should, therefore, be included in the follow-up of borderline lesions.

### Approval 100%

Visualization of tumor vascularization at CEUS is the direct result of the use of a contrast agent with intravascular distribution, a dynamic observation of the post-contrast phase, high spatial resolution, and of the current US contrast harmonic imaging. The ability of this method to cancel all signals coming from the background, so that the operator can see on the monitor only the intensity of the signal produced by the contrast agent passing under the US probe, while the non-vascularized tissues are not depicted, can easily be exploited in the evaluation of the walls and structure of pancreatic cystic lesions. The vascularized vital portions of pancreatic cystic tumors become progressively echoic during CEUS, while the contrast medium is passing through the capillary bed of the septa or the nodules inside the cysts. Intralesional debris, mucus, or blood clots, which are easily visible at the conventional US, are completely invisible in the post-contrast phase [8, 24]. For this reason, CEUS is reported to improve the characterization of pseudocysts [8, 24]. Moreover, due to the deletion of the underlying tissues and intracystic echoic contents (such as the mucinous contents), the detection rate of septa and nodules at CEUS is higher [22] than that of conventional US. During the conventional US, the viscosity of the mucin inside the lesion causes increased echogenicity, which may mask the internal wall thus leading to an incorrect diagnosis [8, 24]. The difference in diagnostic accuracy between CEUS and MRI in identifying septa and nodules is not significant. The data reported in the literature suggest that all patients with asymptomatic pancreatic cystic lesions without signs of suspected malignancy should be monitored. On the basis of these results, we believe that after an initial complete imaging assessment of a pancreatic cystic mass, the conventional US can be used as a follow-up method in lesions that do not require surgery. If changes are observed at the conventional US follow-up, CEUS can be performed. This would limit the use of MRI and CT, thereby reducing exposure to ionizing radiation and the costs. Data reported by D'Onofrio et al. showed that the results of CEUS and MRI in detecting intralesional septa and nodules are very similar in pancreatic cystic masses visible at abdominal US examination. CEUS should be considered a complementary imaging tool in the characterization of pancreatic cystic masses detected at abdominal conventional US and should, therefore, be included in the monitoring of borderline lesions. In the subgroup of patients whose cystic masses can be visualized at US, CEUS could be a less expensive imaging method, free of ionizing radiation, and effective in the monitoring of the lesions [25].

As regards the use of CEUS in pancreatic pathologies, the most recent update of the EFSUMB guidelines was drawn up in 2017 and published in 2018 [26].

## Invasive diagnostics/US-guided needle aspiration/biopsy

Recommended guidelines INVUS (Guidelines on Interventional Ultrasound) [27]

1. In patients with resectable pancreatic masses and typical imaging features of pancreatic ductal adenocarcinoma, no preoperative sampling should be carried out and the patients should be referred directly to surgical evaluation (LoE 2b, GoR B).
2. Resectable pancreatic masses with atypical imaging features should be referred to EUS evaluation and EUS-guided sampling (LoE 3b, GoR B).
3. Borderline resectable pancreatic masses candidates for neoadjuvant therapy should be referred to EUS evaluation and EUS-guided sampling (LoE 2b, GoR C).
4. Locally advanced, unresectable solid pancreatic masses in patients who are candidates for cancer therapy should be referred to diagnostic biopsy (LoE 2b, GoR B).
5. Locally advanced, unresectable pancreatic masses should be evaluated for US-guided percutaneous biopsy. If percutaneous approach is not feasible, EUS approach should be considered (LoE 5, GoR D).
6. Percutaneous US-guidance should be preferred to CT guidance because of the lower complication rates (LoE 2b, GoR B).
7. Biopsy should be performed on suspected liver metastases, if any, to establish a diagnosis and staging of the disease (LoE 5, GoR D).
8. Tissue sampling of pancreatic cystic masses should be performed under EUS guidance (LoE 5, GoR D).
9. Pancreatic cystic masses with typical imaging features requiring surgical treatment should not be sampled before resection (LoE 5, GoR D).
10. US-guided percutaneous biopsy of a transplanted pancreas must be carried out in a transplant center (LoE 5, GoR D).

### Approval 100%

- Percutaneous FNA carried out under US guidance is a sensitive, accurate, and safe procedure in the diagnosis and management of solid pancreatic neoplasms. Diagnostic accuracy reported in the literature (98.7%) is based on a high number of cases [28].
- The presence of a cytologist and the use of suction needles permit acquisition of samples of high diagnostic quality, thus reducing the need to repeat FNA [28].

### Approval 100%

Results obtained in large series reported in the literature reveal that percutaneous FNA of pancreatic masses present a diagnostic accuracy ranging between 75 and 99.4% [28–30]. Considering only FNA carried out under US guidance, a diagnostic accuracy ranging between 91 and 99.4% has been reported with sensitivity ranging between 81 and 99.4% [28–30]. The results of the study carried out by D'Onofrio et al. thus compare favorably with the data reported in the literature in terms of sensitivity and diagnostic accuracy (98.7%), but they are obtained in a larger patient population. Percutaneous FNA performed under US guidance is a sensitive, accurate, and safe procedure in the diagnosis and management of solid pancreatic neoplasms. The data related to the study carried out by D'Onofrio et al. support the use of percutaneous US-guided FNA compared to EUS guidance or biopsy as the first-line tool for invasive characterization of unresectable solid pancreatic lesions. The presence of a cytologist and the use of suction needles permit acquisition of samples of high diagnostic quality, thus reducing the need to repeat FNA [28].
